# Cloning and Characterization of Sf9 Cell Lamin and the Lamin Conformational Changes during *Autographa californica multiple nucleopolyhedrovirus* Infection

**DOI:** 10.3390/v8050126

**Published:** 2016-05-07

**Authors:** Wenqiang Wei, Hongju Wang, Xiaoya Li, Na Fang, Shili Yang, Hongyan Liu, Xiaonan Kang, Xiulian Sun, Shaoping Ji

**Affiliations:** 1 Laboratory of Cell Signal Transduction, Medical School, Henan University, Kaifeng 475004, China; weiwq168@163.com (W.W.); wang_hj168@163.com (H.W.); nanakf@163.com (N.F.); 15903655189@163.com (H.L.); 2 School of Education Science, Henan University, Kaifeng 475004, China; hnu_lxy@163.com (X.L.); k872193085@163.com (X.K.); 3 Key Laboratory of Agricultural and Environmental Microbiology, Wuhan Institute of Virology, Chinese Academy of Sciences, Wuhan 430071, China; yangsl@wh.iov.cn

**Keywords:** *Autographa californica multiple nucleopolyhedrovirus*, infection, Sf9 cells, lamina

## Abstract

At present, the details of lamina alterations after baculovirus infection remain elusive. In this study, a *lamin* gene in the Sf9 cell line of *Spodoptera frugiperda* was cloned. The open reading frame (orf) of the Sf9 *lamin* was 1860 bp and encoded a protein with a molecular weight of 70 kDa. A transfection assay with a red fluorescence protein (rfp)-lamin fusion protein indicated that Sf9 lamin was localized in the nuclear rim. Transmission electron microscopy observations indicated that *Autographa californica multiple nucleopolyhedrovirus* (AcMNPV) nucleocapsids may pass through the nuclear envelope. Immunofluorescence assay indicated that the lamina showed a ruffled staining pattern with the formation of invaginations in the Sf9 cells infected with AcMNPV, while it was evenly distributed at the nuclear periphery of mock-infected cells. Western blotting results indicated that the total amount of lamin in the baculovirus-infected Sf9 cells was significantly decreased compared with the mock-infected cells. These results imply that AcMNPV infection induces structural and biochemical rearrangements of lamina of Sf9 cells.

## 1. Introduction

The nuclear membrane consists of the outer and inner nuclear membranes separated by the perinuclear space, the nuclear pore complexes, and the nuclear lamina. The nuclear lamina lines the nucleoplasmic face of the inner nuclear membrane (INM). It confers structure and mechanical strength to the nuclear membrane and provides attachment sites for chromatin. The lamina forms a thick and rigid meshwork of proteins that is involved in many cellular functions, such as nuclear assembly, cell mitosis, DNA replication, and transcription [1,2].

Lamins are members of the intermediate filament (IF) protein family, which is composed of a central coiled-coil domain flanked by an amino terminal “head” and a carboxyl “tail” domain [3]. Lamins can be classified as A-type or B-type in vertebrate cells, based on their structural features, expression levels, and localization patterns [4]. All lamin proteins, except lamin C, have a CaaX (C is cysteine, a indicates an aliphatic amino acid, X is variable) isoprenylation motif in their carboxy-terminal, which is subject to post-translational modification [3]. The B-type lamins are associated with the nuclear membrane during mitosis and are expressed in almost all cell types, whereas the A-type lamins become soluble during mitosis and are expressed only in differentiated cells. Moreover, lamins also contain a nuclear localization signal (NLS) and cdc2 phosphorylation sites [5]. Although the lamina has been comprehensively studied in vertebrate cells, the information on the lamina of invertebrates is limited. *Drosophila melanogaster* has both lamin Dm0 and lamin C, but lamin C is unique to *Drosophila melanogaster* [6]. It is currently thought that almost all invertebrates have a single B-type lamin, except for *Tunicates* and *Drosophila melanogaster* [5,7].

Herpesvirus infection has been shown to result in structural and biochemical rearrangements of the lamina that allow for viral egress [8,9,10,11]. The UL31 and UL34 protein complex of *human herpesvirus 1* (HHV-1) can disrupt the lamina to promote nucleocapsid egress from the nucleus [8]. HHV-1 recruits cellular protein kinase C to phosphorylate emerin and lamin to induce the disruption of nuclear lamina [9]. Additionally, the kinase UL97 in human cytomegalovirus (HCMV) phosphorylates lamin A/C to reconstruct the lamina [10]. Similarly, the UL50 and UL53 of HCMV remodel the nuclear lamina to allow for the exit of virions from the nucleus [11].

A baculovirus is an enveloped, double-stranded DNA virus, which produces two types of virions: budded viruses (BVs) and occlusion body-derived viruses (ODVs) [12]. BVs mediate the viral spreading between insect tissues or cells. The nucleocapsids of progeny virions are assembled in the nucleus and exit from the nucleus. The most widely accepted model for BV nuclear egress suggests that nucleocapsids leave the nucleus through budding events at the nuclear envelope [13]. Transmission electron microscopy showed that the nucleocapsids in the nucleus align with the INM and enter the perinuclear space by budding through the INM [14]. Wheat germ agglutinin-gold labeling experiments demonstrated that nucleocapsids move from the prominent pore in the nuclear membrane to the cytoplasm [15]. These data provide evidence that baculoviruses may pass through the nuclear membrane and then enter the cytoplasm. Recently, it has been shown that the deletion of open reading frame (orf) 141, orf66, or orf93 of the model baculovirus *Autographa californica multiple nucleopolyhedrovirus* (AcMNPV) led to a disability in nucleocapsid egress [16,17,18].

The nuclear lamina attached to the INM may become a barrier to release of nucleocapsids from the nucleus. Although it seems likely that baculoviruses pass through the lamina during viral egress, it is unknown whether or how the lamina is modified during baculovirus infection. In this study, we cloned the orf sequence of lamin (similar to the *Drosophila melanogaster* nuclear lamin Dm0) in Sf9 cells and observed some of the changes in Sf9 lamin following baculovirus infection.

## 2. Materials and Methods

### 2.1. Cells and Virus

Sf9 cells were cultured at 27 °C in Grace’s medium (Invitrogen, Carlsbad, CA, USA) supplemented with 10% fetal bovine serum (Gibco, Grand Island, NY, USA). The baculovirus vAcBac has been described previously [19].

### 2.2. Reverse Transcriptase Polymerase Chain Reaction (RT-PCR)

Total intracellular RNAs were isolated from Sf9 cells (3.0 × 10^6^ cells/flask) by TRIZOL reagent (Invitrogen). The extracted RNA samples were treated with RNase-Free DNase I (TaKaRa Biotechnology Co. Ltd., Dalian, China) to remove the possible genomic DNA. The first-strand cDNA was synthesized using reverse transcriptase (Invitrogen) and adaptor primer (AP) (GCTGTCAACGATACGCTACGTAACGGCATGACAGTGTTTTTTTTTTTTTTTTTT) with 2 μg total RNA as template. The sequence of *Bombyx mori lamin* was used to search homologues in Sf9 cell against the SPODOBASE database [20]. Sf9 lamin specific primer pairs, *lamin*-orf-F (CGCGGATCCATGTCGTCAAAAACGAAAAAG) and *lamin*-orf-R (GCTCTAGATTACATGATAC GACAGTTCTCTTC) (*Bam*HI and *Xba*l sites, respectively, are underlined), were designed based on two expressed sequence tags that share a high degree of similarity with of *Bombyx mori lamin*. The cDNA mixtures were amplified by KOD polymerase (Toyobo, Osaka, Japan) using the primers *lamin*-orf-F and *lamin*-orf-R. The purified PCR products were cloned into pMD-19T vector (TaKaRa) to obtain *lamin*-T. The *lamin*-T was sequenced (Sangon, Shanghai, China). The alignment of *lamin* nucleotide and protein sequence of Sf9 cells with that of other species was carried out by the program Multalin [21]. The identity of lamin nucleotide and amino acids of Sf9 with its homologues was analyzed by using EMBOSS needle [22]. The coils program was used to predict the coiled-coil domain [23]. The phosphorylation sites recognized by cdk2 kinase were predicted based on the previous study [24]. The Predictprotein server was used to predict the NLS [25].

The red fluorescence protein (rfp) gene, amplified from pDsRed2-N1 (Clontech, Palo Alto, CA, USA) with the primers *rfp*-F (CCC AAGCTTATGGCCTCCTCCGAGAACGT) and *rfp*-R (CGCGGATCCCAGGAACAGGTGGTGGCGG) (*Hin*dIII and *Bam*HI sites, respectively, are underlined), was digested with *Hin*dIII and *Bam*HI and ligated to pIZ-V5/his vector (Invitrogen), downstream of the OpMNPV IE2 promoter, to generate piz-*rfp*. The *lamin*-T was digested with *Bam*HI and *Xba*l and cloned into piz-*rfp* to generate the piz-*rfp*-*lamin*.

### 2.3. Transmission Electron Microscopy

Sf9 cells (1.0 × 10^6^ cells/35-mm-diameter plate) were infected with vAcBac at a multiplicity of infection (MOI) of 5. At various time points post-infection (p.i.) (12, 24, 48 h p.i.), the cells were pelleted, fixed in 2.5% glutaraldehyde overnight at 4 °C, washed three times with 0.1 M PBS (pH 7.2), followed by fixation with 1% osmium tetroxide for 3 h at room temperature. After dehydration through a graded ethanol (30%–100%), cells were embedded in spur resin. After staining with uranyl acetate and lead citrate, the ultrathin sections were examined under a Hitachi H-800 transmission electron microscope (Hitachi Co., Ltd., Tokyo, Japan).

### 2.4. Transfection

Sf9 cells (1.0 × 10^6^ cells/35-mm-diameter plate) were transfected with 2.0 μg plasmid piz-*rfp*-*lamin* using 8 μL lipofectamine reagent (Invitrogen) according to the manufacture’s instruction. After incubation for 5 h, the transfection supernatants were discarded and the cells were replenished with 2 mL fresh grace’s medium supplemented with 10% fetal bovine serum, 100 μg/mL of penicillin and 30 μg/mL of streptomycin. The nucleus was stained with Hoechst 33258 (blue) at 48 h post-transfection (h p.t.). The fluorescence was observed with a Zeiss confocal microscope (Zeiss, Oberkochen, Germany).

### 2.5. Immunofluorescence

Sf9 cells (1.0 × 10^6^ cells/35-mm-diameter plate) were infected with vAcBac at a MOI of 5. At various time points post-infection, the cells were washed three times in PBS, and fixed with 4% paraformaldehyde for 10 min at room temperature. The cells were washed three times in PBS, permeabilized with 0.5% Triton X-100 in PBS for 10 min. After washing three times with PBS, the cells were blocked in 3% BSA for 1 h, incubated with anti-Lamin antibody DL67 (diluted 1:10 in blocking solution, provided by P. A. Fisher, Department of Pharmacological Sciences, University of New York at Stony Brook) at 4 °C overnight. The cells were rinsed three times with PBS and incubated with the tetramethylrhodamine isothiocyanate-dextran (TRITC)-labeled anti-mouse IgG (1:100, ZSGB-BIO, Beijing, China) for 2 h. The cells were washed three times with PBS, stained with Hoechst 33258 for 10 min, rinsed three times with PBS, and observed with a Zeiss confocal microscope.

### 2.6. Western Blotting

Sf9 cells (1.0 × 10^6^ cells/35-mm-diameter plate) were infected with vAcBac at an MOI of 5. The cells were harvested at different time points, centrifuged at 10,000 g for 10 min. The pellets were suspended in 6× SDS-PAGE loading buffer (Transgen, Beijing, China) and boiled for 10 min. Protein samples were separated by sodium dodecyl sulfate polyacrylamide gel electrophoresis (SDS-PAGE), transferred onto a polyvinylidene fluoride (PVDF) membrane. The membrane was blocked in 5% skimmed milk for 1 h. The immunoreactive proteins were detected using anti-lamin ADL67 (1:50) or anti-actin antibody (1:1000; Sangon Biotech, Shanghai, China) incubated at 4 °C overnight. After washing, the membrane was incubated with anti-mouse or anti-rabit IgG conjugated with horseradish peroxidase (1:3000, Boster, Wuhan, China) for 2 h. The signal was visualized by enhanced chemiluminescence system (Amersham). Quantification of western blotting analysis for change in the amount of lamin was performed by Image-Pro Plus 5.0 software (Media Cybernetics, Silver Spring, MD, USA). The total amount of lamin at 24, 48, 72, 96 h p.i. was compared with those in mock-infected cells by one-way ANOVA followed by Dunnett *t* tests.

## 3. Results and Discussion

**Figure 1:**
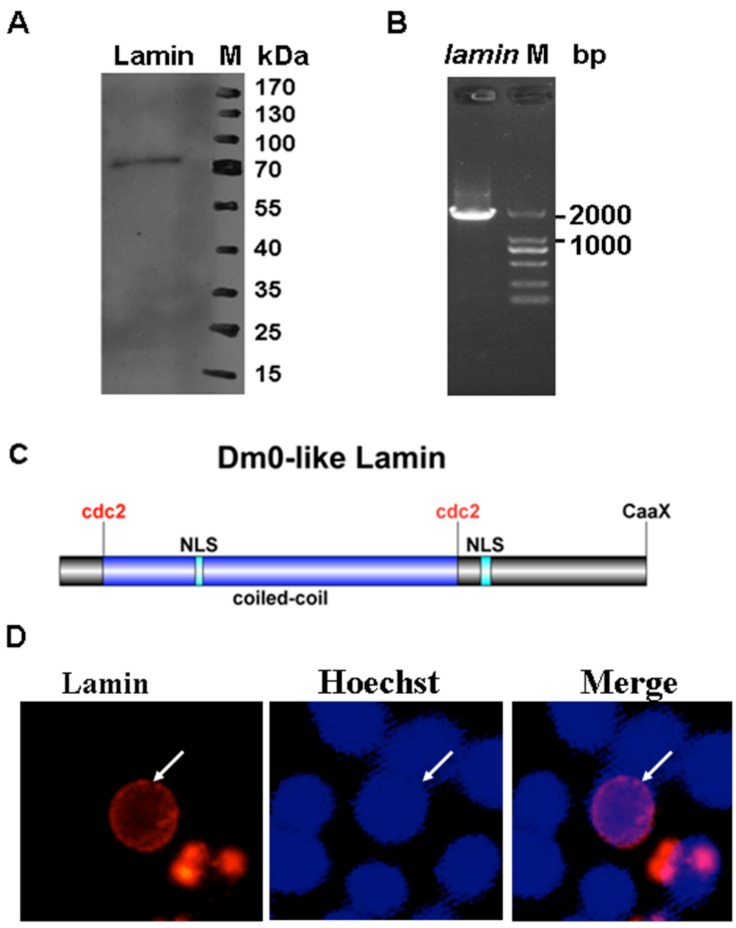
The expression and bioinformatic analysis of lamin in Sf9 cells. (**A**) The total protein was extracted from Sf9 cells, subjected to sodium dodecyl sulfate polyacrylamide gel electrophoresis (SDS-PAGE), and transferred to a polyvinylidene fluoride (PVDF) membrane. The protein was detected with an anti-Dm0 monoclonal antibody, ADL67, and the bound antibody was detected with an HRP-labeled secondary antibody. The protein sizes are indicated on the right. (**B**) Total RNAs were isolated from Sf9 cells, and the RNA samples were treated with RNase-free DNase I. The first-strand cDNA was synthesized by reverse transcription using 2 μg total RNAs as template. The cDNA mixtures were amplified by KOD polymerase using *lamin*-specific primers. The approximate molecular size in bp is shown. (**C**) The predicted structure of Sf9 cell lamin. The lamins have a central coiled-coil domain (blue box) flanked by short head and long tail domains. The coiled-coil domain is flanked by cdc2 phosphorylation sites. Two NLSs were predicted in the coiled-coil domain and the tail domain. The CaaX motif is in the C-terminal. (**D**) Sf9 cells were transfected with pIZ-*rfp*-*lamin* plasmids to show the sub-cellular distribution of lamin. The nucleus was stained with Hoechst 33258 (blue) at 48 h p.t., and the lamin expression (red) was observed by fluorescence microscopy.

To determine whether the B-type lamins exist in Sf9 cells, Western blotting assays were performed using the anti-Dm0 antibody, ADL67 and the anti-mouse secondary antibody. A 70 kDa protein band was observed from the total protein lysates of Sf9 cells (Figure 1A). It has been found that the molecular weight of nuclear lamin lies between 60 and 80 kDa [7]. The lamin of Sf9 cells is indistinguishable in its molecular weight from *Drosophila* lamin Dm0, suggesting that these Sf9 cells do express the lamin. A 1851 bp fragment was amplified from the Sf9 cell-derived cDNA mixtures by RT-PCR using the primer pairs (*lamin*-orf-F/R) and sequenced by using an Illumina MiSeq desktop 316 sequencer (Figure 1B). The orf of Sf9 *lamin* (GenBank accession number: KT318393) is predicted to code a protein of 616 amino acids with a molecular weight of 70 kDa, consistent with the result of the above-mentioned Western blotting.

Bioinformatics analysis was performed to predict the characteristics of Sf9 lamin. The Coils program [23] predicted the lamin in Sf9 cells has a central coiled-coil domain at 46–418 aa residues, which may mediate the dimerization of the lamin protein (Figure 1C). A CaaX motif was found at its C-terminus (Figure 1C). The phosphorylation prediction found two sites that are recognized by cdc2 kinase (47 and 418 aa; Figure 1C). The Predict Protein program [25] predicted that the lamin in Sf9 cells contains two NLS motifs (143–151 and 442–453 aa; Figure 1C). These data indicate that the lamin contains structural motifs that are similar to the classic lamina of mammalian cells. A comparison between the amino acids that compose lamins from several sources shows that the lamin in Sf9 cells has 92% identity with *Bombyx mori* lamin, while it is 20%–50% identical to the homologous proteins of other species (Table 1).

The vertebrate lamina is enclosed in the INM. To observe the cellular localization of Sf9 cell lamin, the plasmid pIZ-*rfp*-*lamin* was constructed in which the Sf9 cell *lamin* orf fused with the red fluorescence protein gene was cloned into a pIZ-V5/His vector. The transfection assay indicated that lamin was distributed around the nuclear rim of Sf9 cells (Figure 1D, solid arrow). These features, combined with the above biochemical and bioinformatics results, support the conclusion that Sf9 lamin is classified as a B-type lamin.

**Figure 2:**
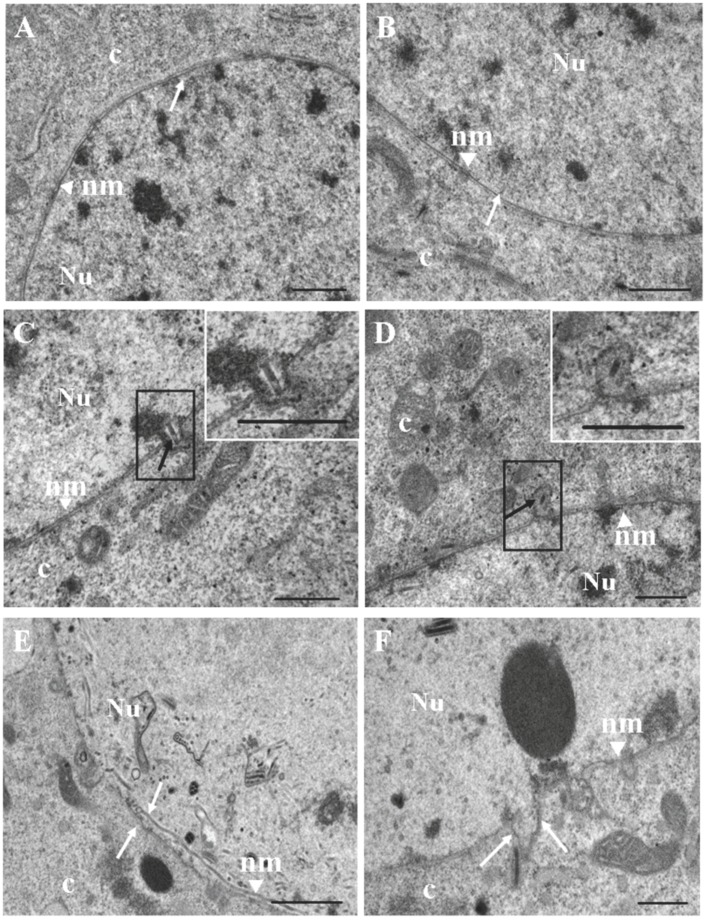
Electron microscopy of vAcBac-infected Sf9 cells. Panels (**A**) and (**B**) show the intact nuclear membrane in mock-infected cells and cells at 12 h post-infection (p.i.), respectively. The white arrows indicate the nuclear membrane. Panels (**C**) and (**D**) show the egress of nucleocapsids from the nuclear membrane at 24 h p.i. Inset shows the boxed region at higher magnification. The black arrows indicate the egress of progeny viral nucleocapsids from the nuclear membrane. Panel (**E**) and (**F**) show the remodeled nuclear membrane at 48 h p.i.. The white arrows indicate variation within the nuclear membrane. Bar, 500 nm (A,B,C,D,F), and 1000 nm (E). Nu, nucleus; c, cytoplasm; nm, nuclear membrane.

To examine whether baculovirus infection induces the disruption of nuclear membrane, transmission electron microscopy observations were performed in Sf9 cells infected by vAcBac or mock-infected. The results indicated that the nuclear membrane was intact and smooth at 12 h p.i. compared with the mock-infected cells (Figure 2A,B, white arrow); however, at 24 h p.i., the nuclear membrane invaginated into the cytoplasm, and the progeny virus nucleocapsids oriented toward the invagination regions, suggesting that the nucleocapsids may pass through the nuclear membrane (Figure 2C,D, black arrow). Interestingly, the outer and inner nuclear membranes appeared to be separated and broken in the circumscribed region at 48 h p.i. (Figure 2E,F, white arrow). It has been previously shown that both the mechanical tearing of the lamina and the biochemical modification of lamin B1 filaments are required for nuclear membrane breakdown [26,27]. We propose that the lamin proteins in Sf9 cells may be involved in the partial disintegration of the nuclear membrane. Taken together, these results imply that baculovirus nucleocapsids may pass through the lamina before arriving at the nuclear membrane.

**Figure 3:**
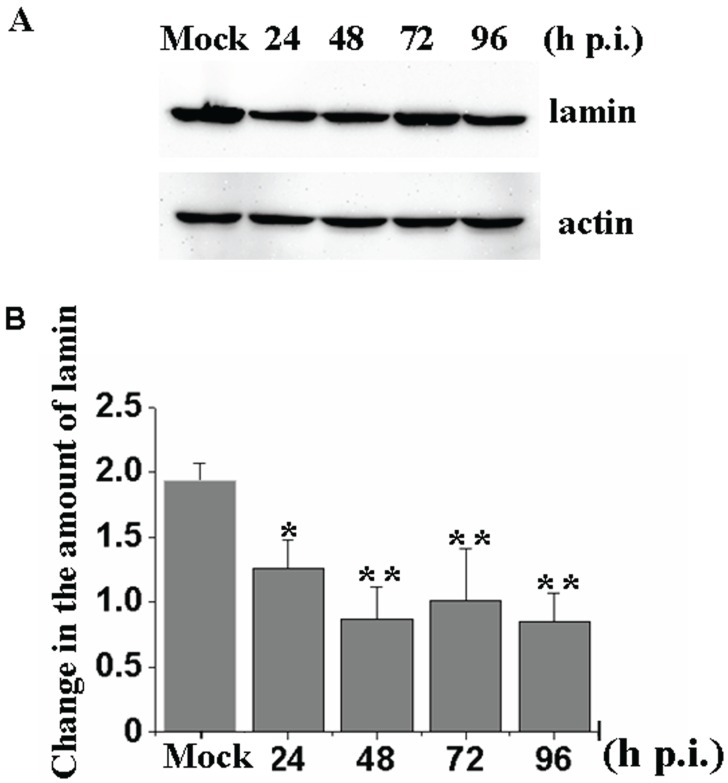
Examination of the amount of lamin during AcMNPV infection. (**A**) Sf9 cells were infected with vAcBac at amultiplicity of infection (MOI) of 5. At various time points (24, 48, 72, 96 h p.i.), the cells were harvested, and the proteins were separated on a 10% polyacrylamide gel and analyzed by western blotting using the anti-Dm0 monoclonal antibody, ADL67, or anti-actin antibody, and the bound antibody was detected with the HRP-labeled secondary antibody. (**B**) Western blotting analysis of changes in the amount of lamin during baculovirus infection. Amounts of lamin were determined by measuring of band densities using Image-Pro Plus 5.0 software. Obtained values were adjusted to actin levels. Mean values (± SE) of lamin protein levels of three different experiments are shown. The Dunnett *t* tests were used to compare the difference between the total amount of lamin in vAcBac-infected cells at 24, 48, 72, 96 h p.i. with those in the mock-infected cells (*p < 0.05, ** *p* < 0.01) after an *F* test.

To determine whether the total amount of lamin in Sf9 cells changes during baculovirus infection, vAcBac-infected cells were examined by Western blotting assays using the ADL67 antibody. The results indicate that throughout the progression of viral infection (24, 48, 72, 96 h p.i.), the total amount of lamin was significantly decreased compared with the amount in mock-infected cells (*F* = 9.163, *p* = 0.002) (Figure 3A,B).

**Figure 4:**
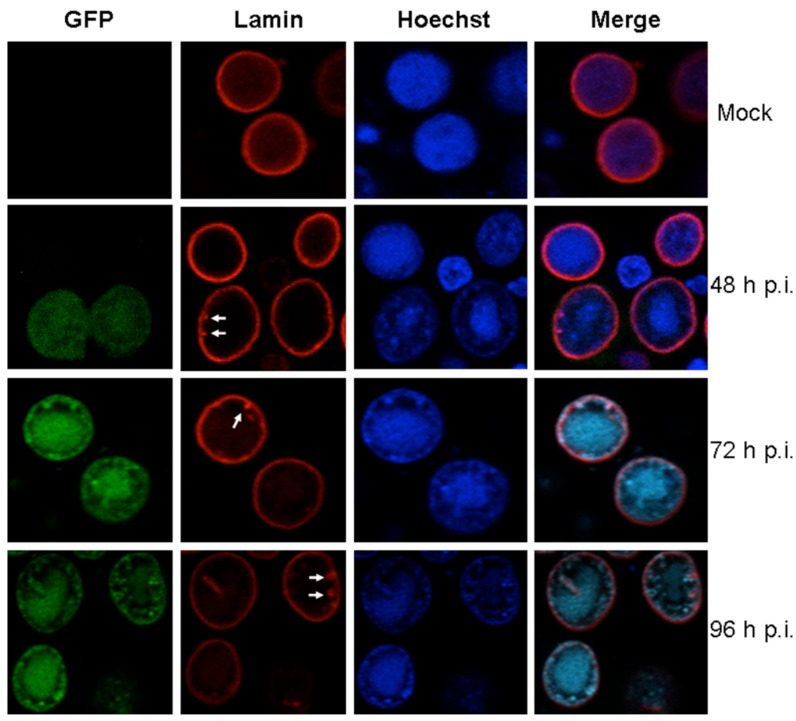
The conformational changes of nucleus lamin during AcMNPV infection. Sf9 cells were infected or mock-infected with vAcBac (expressing GFP) at an MOI of 5 and then fixed in 4% paraformaldehyde. The fixed cells were stained using the anti-Dm0 monoclonal antibody, ADL67, and visualized using a tetramethylrhodamine isothiocyanate-dextran (TRITC) labeled anti-mouse IgG (red). The nucleus was stained with Hoechst 33258 (blue). The white arrows indicate that the lamina showed a ruffled staining pattern with the formation of invaginations in Sf9 cells infected with *Autographa californica multiple nucleopolyhedrovirus* (AcMNPV).

Baculovirus infection induces the formation of intranuclear microvesicles in the nucleus. During the late phase of infection, the nucleocapsids localized in the ring zone are enveloped in the intranuclear microvesicles to form the occlusion body-derived viruses [13]. The AcMNPV ac93 and ac76 proteins may be required for the formation of intranuclear microvesicles [18,28]. Currently, the composition and structure of these vesicles is unclear, but the intranuclear microvesicles are thought to be derived from the INM [29]. Considering the fact that the nuclear lamina is associated with the INM, we cannot exclude the possibility that the nuclear lamin may also be implicated in the formation of microvesicles. Furthermore, invertebrate lamins are involved in chromatin condensation and the repression of gene expression [5]. The Hoechst 33258 can bind to chromatin *in situ* and so was used in this study to stain the nucleus [30]. In Sf9 cells that are infected with vAcBac, the chromatin stained by Hoechst 33258 appeared to be condensed and degraded (Figure 4). A previous study also found that baculovirus infection leads to a shutdown of protein synthesis for the majority of host proteins [31,32,33]. It is necessary to determine whether the changes in the chromatin properties and the decrease in protein synthesis are related to the changes in the nuclear lamina.

To monitor whether baculovirus infection alters the lamina distribution in Sf9 cells, immunofluorescence assays were performed. The results indicate that, in Sf9 cells infected with vAcBac, the lamin had a round or ovoid shape at the nuclear periphery (Figure 4). The obvious disruption of lamin, like that during herpesvirus infection, was not observed till at late stage of infection (96 h p.i). Interestingly, the lamina in Sf9 cells showed a ruffled staining pattern with the formation of invaginations (Figure 4, solid arrow). However, in mock-infected cells, the lamina was evenly distributed at the nuclear periphery without the formation of invaginations (Figure 4). As we know, the cell infection cycle of baculoviruses ends with cell death at approximately 72 h p.i., which may be result from virus-induced cell apoptosis. We presume that the observed invaginations of lamina may be due to the apoptotic process induced by AcMNPV. Taken together, these data indicate that baculovirus infection may have certain influence on the morphogenesis of lamin in the nucleus of Sf9 cells.

From the above results, it has been found that Sf9 cells reveal the morphologic distortion and the significant decrease in the amount of the lamins during baculovirus infection. However, the lamina did not undergo remarkable structural alternations, differing from what have been observed during herpesvirus infection. This implies that the nucleocapsids of AcMNPV may gain access to the INM by the intricate ways. Firstly, baculovirus may pass through the lattice of lamina of insect cell directly and then arrive at the INM. Goldberg *et al.* found that the distance between associated lamin filamenets of *Xenopus* oocyte is approximately 15–16 nm [25]. The diameter of rod-shaped baculovirus budded virus is approximately 40–50 nm. The lattice of the lamina of Sf9 cells may be relatively large and the rod shaped nucleocapsids have access to traverse it. Secondly, the lamin protein may be not homogeneously distributed at the INM in virus-infected Sf9 cells, which is favorable for the nucleocapsids to pass through the lamina. These possibilities will be verified in our future experiments.

In conclusion, the *lamin* gene exists in Sf9 cells and AcMNPV infection results in the significant decrease of the amount of lamin and the formation of invaginations at the nucleus rim. This study may lay the foundation for research on the mechanism of baculovirus nucleocapsid passing through the nuclear membrane and the formation of intranuclear microvesicles.

## Figures and Tables

**Table 1:** Comparison of the deduced amino acid sequence of *lamin* from Sf9 cells with the sequences from Bombyx (*Bombyx mori*, GI: 512922266), Drosophila (*Drosophila melanogaster*, GI: 667674288), Mus (*Mus musculus*, GI: 15929760), Homo (*Homo sapiens*, GI: 224901), and Xenopus (*Xenopus laevis*, GI: 156119432).

	Amino Acid Identity (%)				
	Sf9	Bombyx	Drosophila	Mus	Homo
Bombyx	92				
Drosophila	50	50			
Mus	33	33	31		
Homo	31	30	29	78	
Xenopus	20	20	18	41	34
